# To bind or not to bind: how AUXIN RESPONSE FACTORs select their target genes

**DOI:** 10.1093/jxb/erad259

**Published:** 2023-07-11

**Authors:** Juriaan Rienstra, Jorge Hernández-García, Dolf Weijers

**Affiliations:** Laboratory of Biochemistry, Wageningen University, Stippeneng 4, 6708WE Wageningen, The Netherlands; Laboratory of Biochemistry, Wageningen University, Stippeneng 4, 6708WE Wageningen, The Netherlands; Laboratory of Biochemistry, Wageningen University, Stippeneng 4, 6708WE Wageningen, The Netherlands; Institute of Science and Technology Austria (ISTA), Austria

**Keywords:** Auxin, Auxin Response Element, AuxRE, Auxin Response Factor, dimerization, DNA binding, gene expression, transcription, transcription factors

## Abstract

Most plant growth and development processes are regulated in one way or another by auxin. The best-studied mechanism by which auxin exerts its regulatory effects is through the nuclear auxin pathway (NAP). In this pathway, Auxin Response Factors (ARFs) are the transcription factors that ultimately determine which genes become auxin regulated by binding to specific DNA sequences. ARFs have primarily been studied in *Arabidopsis thaliana*, but recent studies in other species have revealed family-wide DNA binding specificities for different ARFs and the minimal functional system of the NAP system, consisting of a duo of competing ARFs of the A and B classes. In this review, we provide an overview of key aspects of ARF DNA binding such as auxin response elements (TGTCNN) and tandem repeat motifs, and consider how structural biology and *in vitro* studies help us understand ARF DNA preferences. We also highlight some recent aspects related to the regulation of ARF levels inside a cell, which may alter the DNA binding profile of ARFs in different tissues. We finally emphasize the need to study minimal NAP systems to understand fundamental aspects of ARF function, the need to characterize algal ARFs to understand how ARFs evolved, how cutting-edge techniques can increase our understanding of ARFs, and which remaining questions can only be answered by structural biology.

## Teaser

We discuss the biochemical principles underlying the regulation of gene expression by ARF transcription factors, the output of the auxin response.

## Introduction

**Fig. 1.**
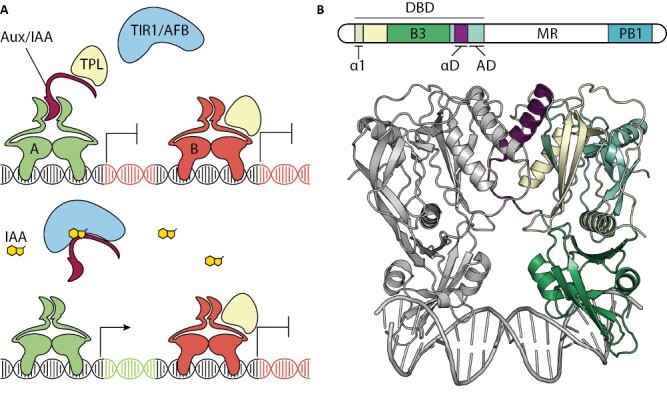
Overview of the nuclear auxin pathway and the anatomy of an ARF. (A) The minimalistic nuclear auxin pathway. Under low auxin conditions (upper panel), both A-ARFs (green) and B-ARFs (red) act as repressors. A-ARFs bind with a repressive cofactor, Aux/IAA (bordeaux), that recruits TPL, while B-ARFs recruit TPL directly via their middle region. Under increasing auxin conditions [indole-3-acetic acid (IAA) gold], IAA acts as a molecular glue and allows TIR1/AFB to sequester Aux/IAA away from the A-ARFs, which then become transcriptional activators. B-ARFs act as repressors in either condition. (B) The anatomy of an ARF. Top panel shows the general genetic sequence of an ARF, the lower panel shows the atomic structure of the DNA-binding domain in complex with an IR7 motif (pdb: 6ycq). Domains indicated and discussed in this review are: α1 (pear), the α-helix tethering the B3 domain and acting as a molecular hinge; N-terminal dimerization domain (yellow); B3 (green), the domain interacting with the DNA; αD (purple), the α-helix and loop that facilitates dimerization; and the C-terminal dimerization domain and ancillary domain (AD, cyan). The middle region (MR, white) and Phox and Bem1 domain (PB1, blue) are omitted.

Auxin is a signaling molecule involved in virtually every plant developmental process (Weijers and Wagner, 2016). Most responses triggered by auxin are mediated by the nuclear auxin signalling pathway (NAP) (Fig. 1). The NAP is composed of three auxin-specific elements: the TRANSPORT INHIBITOR 1/AUXIN SIGNALING F-BOX (TIR1/AFB) family of receptors, the AUXIN/INDOLE-3-ACETIC ACID (Aux/IAA) family of co-repressors, and the AUXIN RESPONSE FACTOR (ARF) family of DNA-binding transcription factors. Auxin acts as a molecular glue to promote interactions between TIR1/AFB and Aux/IAA proteins, leading to the ubiquitination and subsequent destabilization of the latter (Tan *et al.*, 2007). Aux/IAA proteins act as transcriptional co-repressors by directly interacting with ARFs and imposing transcriptional repression (Ulmasov *et al.*, 1997b; Tiwari *et al.*, 2001). Upon Aux/IAA degradation, ARFs are released to regulate gene expression (Tiwari *et al.*, 2001, 2003). In the end, the ARFs are the ultimate arbiters that decide which genes become auxin regulated by virtue of their sequence-specific DNA binding (Ulmasov *et al.*, 1997b).

ARF proteins contain three conserved domains with distinct functions: an N-terminal DNA-binding domain (DBD), a middle region (MR), and a C-terminal Phox and Bem1 (PB1) domain (Fig. 1) (Ulmasov *et al.*, 1999b; Tiwari *et al.*, 2003; Weijers and Wagner, 2016). The DBD contains a B3 domain that allows ARFs to bind DNA motifs called Auxin Response Elements (AuxREs) (Ulmasov *et al.*, 1999b; Boer *et al.*, 2014; Weijers and Wagner, 2016). Other regions within the DBD facilitate homodimerization and heterodimerization, imposing an additional layer of DNA binding preference towards tandem repeat AuxREs (see further) (Ulmasov *et al.*, 1997a, b; Boer *et al.*, 2014). Once bound to chromatin, ARFs can either repress or activate transcription, through their MR (Ulmasov *et al.*, 1999a; Kato *et al.*, 2015). The PB1 domain acts as a homo- and heterotypic oligomerization domain, allowing ARFs to homo- and/or heterooligomerize and/or interact with Aux/IAA proteins (Kim, 1997; Ouellet *et al.*, 2001; Hamann *et al.*, 2002; Li *et al.*, 2011; Kato *et al.*, 2015).

ARFs probably originated in a streptophyte common ancestor to land plants and algal sisters, and diverged into three phylogenetically and functionally separated classes present in all subsequent land plants: A, B, and C (Mutte *et al.*, 2018). Extant streptophyte algal ARFs belong either to the C-class, or to an A/B-class that probably represents an ancestral state preceding A/B diversification (reviewed in Kato *et al.*, 2018; Carrillo-Carrasco *et al.*, 2023). While algal ARFs have not been studied in depth, analysis of the single ARF in *Chlorokybus atmophyticus* suggest a DNA binding preference similar to that of land plant counterparts (Martin-Arevalillo *et al.*, 2019). This indicates that the fundamental ARF DNA binding preferences are probably ancestral and have been maintained during evolution. In land plants, ARF transcriptional activities are well conserved between bryophytes and angiosperms, and are attributable to the different ARF classes: A-ARFs are known to act mainly as transcriptional activators, while B- and C-classes function as repressors (Tiwari *et al.*, 2003; Kato *et al.*, 2015). A- and B-class ARFs are known to regulate auxin transcriptional responses in land plants, whereas the integration of C-ARFs into the auxin response network is less clear (Flores-Sandoval *et al.*, 2018a, b; Mutte *et al.*, 2018). Among land plants, the genome of the liverwort *Marchantia polymorpha* (Marchantia) encodes the simplest NAP studied to date, with a single TIR1/AFB, a single Aux/IAA, and a single ARF for each of the three classes (Flores-Sandoval *et al.*, 2015; Kato *et al.*, 2015, 2017, 2020; Bowman *et al.*, 2017; Mutte *et al.*, 2018). In contrast, repeated gene and genome duplications have shaped more complex NAP families in flowering plants, represented by six TIR1/AFB, 29 AUX/IAA, and 23 ARF (5A, 15B, 3C) members in *Arabidopsis thaliana* (Arabidopsis) (Weijers and Wagner, 2016; Mutte *et al.*, 2018). In Marchantia, the single A-ARF and B-ARF (MpARF1 and MpARF2, respectively) compete for the same DNA motifs (Kato *et al.*, 2020), a mechanism shared with representatives of the moss *Physcomitrium patens* A- and B-class ARFs (Lavy *et al.*, 2016). In this model, MpARF1 is an auxin-sensitive transcriptional regulator that switches between Aux/IAA-imposed transcriptional repression in the absence of auxin, and ARF-mediated transcriptional activation in the presence of auxin, while MpARF2 represses independently of auxin and acts to limit auxin responsiveness (Fig. 1). This basic module may reflect the system present in the (elusive) last common ancestor of land plants. However, this module expanded significantly during land plant evolution, leading to an increase in the web of interactions, to redundancy between ARFs, and sub- and neofunctionalization of ARFs (Hardtke *et al.*, 2004; Nagpal *et al.*, 2005; Okushima *et al.*, 2005; Weijers *et al.*, 2005; Li *et al.*, 2006; Rademacher *et al.*, 2011, 2012).

In this review, we discuss large-scale DNA binding assays and promoter studies that have elucidated ARF binding specificities. We will discuss this in the context of ARF DNA binding structural biology, and then briefly focus on other aspects that are likely to affect ARF DNA binding such as oligomerization, cytoplasmic condensate formation, and ARF E3 ligase-mediated degradation. We touch on aspects that add levels of complexity to the basic A/B competition model proposed for Marchantia, and that may underlie the intricate scenario of auxin transcriptional regulation in other land plants.

## Selective ARF binding to Auxin Response Elements

ARFs select their target genes based on the presence of AuxREs, defined as TGTCNN elements (where N can be any nucleotide). One of the first identified AuxREs was the TGTCTC motif of the soybean *GRETCHEN HAGEN 3* (*GH3*) promoter, later used as a ‘bait’ to identify the first ARF: AtARF1 (Liu *et al.*, 1994; Ulmasov *et al.*, 1995, 1997a, 1999b). The TGTC acts as the invariable core element vital for auxin response, while the final two nucleotides are variable (Liu *et al.*, 1994; Ulmasov *et al.*, 1995, 1999b; Boer *et al.*, 2014). Protein binding microarray analyses of Arabidopsis ARFs show that ARFs bind a multitude of TGTCNN elements, each with different affinities, with the highest affinity towards the TGTCGG element (Boer *et al.*, 2014; Franco-Zorrilla *et al.*, 2014). In line with this, a gene expression reporter based on a multimer of TGTCGG has an ~10-fold higher auxin sensitivity than a TGTCTC-based reporter (Liao *et al.*, 2015). Recently, genome-wide ARF DNA binding studies of several maize A- and B-class ARFs indicated that all family members can bind a variety of TGTCNN elements but strongly prefer TGTCGG elements (Galli *et al.*, 2018).

**Fig. 2.**
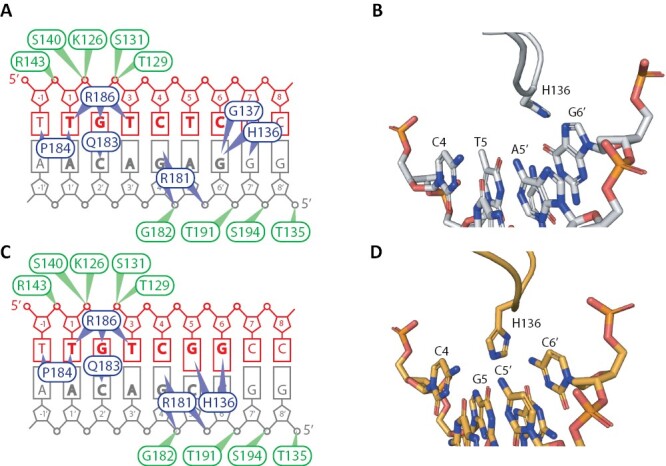
Atomic interface between AuxRE and ARF-B3. Residues are numbered according to AtARF1 and DNA bases are numbered according to the first nucleotide of TGTCTC. (A and C) Schematic representation of DNA contact sites of ARF-B3 for TGTCTC (A) and high affinity TGTCGG (C). Contacting residues are indicated with ovals, green for residues only contacting the phosphate backbone and blue for residues contacting specific DNA bases. The figures are composites of three structures (4ldx, 6ycq, and 6sdg; cut-off for contacts of 3.5 Å) and, as such, some contacts are only found in a single structure, or compensated for by other residues. For instance, either K126 makes contacts alone, or T129 and S140 contact together, but never all three at the same time. (B and D) Close up of AtARF1–H136 conformation change with AuxRE bases of either TGTCTC (B) or TGTCGG (D). Based on Freire-Rios *et al.* (2020).

The mechanism for both the generic ability to bind the TGTC core and the preference for TGTCGG elements can be found in the crystal structure. The DNA-binding residues were first identified in AtARF1 in complex with TGTCTC DNA (4ldx; Boer *et al.*, 2014) (Fig. 1). More recently, the structures of AtARF1 (6ycq; Freire-Rios *et al.*, 2020) and MpARF2 (6sdg; Kato *et al.*, 2020) in complex with TGTCGG DNA was solved. This allowed us to re-examine and update the residues involved in DNA binding using the AtARF1 sequence as reference (Fig. 2). The B3 domain harbours two types of DNA-binding residues, the first of which are residues that interact with the DNA phosphate backbone to confer general DNA binding affinity via hydrogen bonds and electrostatic interactions (Fig. 2A, C). The second type of DNA-binding residues enter the DNA major groove and confer TGTCNN specificity. These are found in two loops: a C-terminal loop of which residues Q183, P184, R181, and R186 bind the core TGTC; and an N-terminal loop where G137 and H136 are involved in binding the two variable residues (Fig. 2A, C). TGTCGG elements are probably bound with higher affinity than TGTCTC because H136 is rotated deeper into the major groove and forms additional hydrogen bonds with G5 and G6 in the TGTCGG structure (Fig. 2C, D). In comparison, in the TGTCTC structure, this histidine is interacting only with G6ʹ and G7ʹ, and is sterically hindered from rotating deeper into the DNA by C6 and G6ʹ (Fig. 2A, B) (Boer *et al.*, 2014; Freire-Rios *et al.*, 2020). It is therefore likely that this H136-containing loop is responsible for the wide variety of TGTCNN elements that ARFs can bind depending on the rotamer and steric hindrances. Given that the general DBD structure and the DNA major groove-binding residues are strongly conserved across ARFs from the plant kingdom (Boer *et al.*, 2014; Mutte *et al.*, 2018; Freire-Rios *et al.*, 2020; Kato *et al.*, 2020), it is likely that TGTCGG elements were the preferential DNA-binding elements for the A- and B-class ancestors. This would also support the notion of A/B competition being an ancestral characteristic of ARF-mediated transcriptional regulation in land plants.

While ARFs show a clear preference for TGTCGG elements, these are not pervasive in auxin-responsive genes and, instead, auxin-responsive promoters have a wide variety of TGTCNN elements (Liu *et al.*, 1994; Xu *et al.*, 1997; Donner *et al.*, 2009; Walcher and Nemhauser, 2012; Zemlyanskaya *et al.*, 2016; Cherenkov *et al.*, 2018; Lieberman-Lazarovich *et al.*, 2019). Still, some of the different TGTCNN elements can be associated with particular modes of auxin response. For example, early auxin-activated genes are associated with TGTCTC elements, such as those found in the *GH3* and *SMALL AUXIN UPREGULATED RNA* (*SAUR*) promoters, while the high-affinity TGTCGG element is found in both early and late auxin-activated and -repressed responses (Zemlyanskaya *et al.*, 2016; Cherenkov *et al.*, 2018; Lieberman-Lazarovich *et al.*, 2019). Elements can also be associated with specific developmental or cellular processes, such as TGTCGG with cell wall-related genes or TGTCTT with phosphate metabolism, the latter of which is shown to probably involve AtARF7 and AtARF19 (Narise *et al.*, 2010; Zemlyanskaya *et al.*, 2016; Huang *et al.*, 2018; Lieberman-Lazarovich *et al.*, 2019). Finally, these elements can be associated with different expression domains. For instance, in the Arabidopsis roots, the same gene expression reporter with different TGTCNN elements has been tested, of which TGTCGG and TGTCTC differ in the location of expression maxima, with TGTCTC reaching a maximum in the quiescent centre, and TGTCGG in the subtending columella cells (Liao *et al.*, 2015). Reporters with TGTCCC, TGTCGC, and TGTCAC show both overlapping and unique expression domains (Lieberman-Lazarovich *et al.*, 2019). These unique domains suggest that they are caused by particular ARFs with alternative DNA binding preferences, but so far it is unknown whether any correlation exists between different TGTCNN reporters and the unique ARF expression domains in the roots (Rademacher *et al.*, 2011; Lieberman-Lazarovich *et al.*, 2019).

## Higher order DNA binding specificity through ARF dimerization

**Fig. 3.**
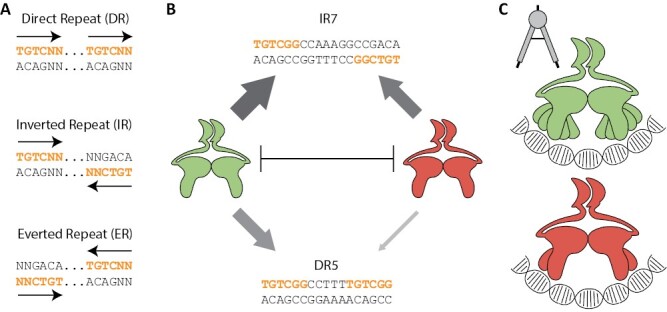
Spacing motifs determine ARF specificity. (A) Definition of a direct repeat (DR), inverted repeat (IR), and everted repeat (ER), following the definition set by Freire-Rios *et al.* (2020). N denotes A, T, C, or G, while dots represent variable numbers of intermittent nucleotides between two TGTCNN elements. (B) Schematic figure representing single-molecule FRET analysis of DNA binding. Arrow thickness represents affinity. A- (green) and B-ARFs (red) are competing with each other for DNA binding. Both ARFs have an affinity for IR7 motifs that allows for *in vivo* competition on the same locus. For DR5, both ARFs have a weaker affinity, with B-ARF affinity being so weak that their *in vivo* binding is likely to be negligible (Freire-Rios *et al.*, 2020; Kato *et al.*, 2020). (C) The proposed caliper model (Boer *et al.*, 2014). The A-ARF AtARF5 (green) was found to be able to bind IR5–IR9 motifs, whereas the B-ARF AtARF1 (red) was limited to IR7/8.

Few studies have identified ARF targets (see below), but those available suggest distinct sets of targets. However, if all ARFs preferentially bind the same element, TGTCGG, it would be difficult to explain how ARFs regulate distinct sets of target genes. Rather, like many eukaryotic transcription factors (Amoutzias *et al.*, 2008; Kribelbauer *et al.*, 2019; Sloan *et al.*, 2020), ARFs bind DNA as dimers, and can homodimerize via their DBD (Boer *et al.*, 2014), and additionally through their PB1 domain (Han *et al.*, 2014; Korasick *et al.*, 2014; Nanao *et al.*, 2014; Kim *et al.*, 2020), to bind tandem repeat motifs of TGTCNN elements. The nomenclature of these tandem repeat is as set by Freire-Rios *et al.* (2020), with inverted repeat (IR), everted repeat (ER), and direct repeat (DR) depending on the orientation of two TGTCNN elements and the number of bases between the individual motifs (Fig. 3A). For example, when two TGTCNN elements point in the same direction (direct repeat) and are spaced five nucleotides apart, this is a DR5 motif. Given that A-ARFs are auxin-dependent activators and B-ARFs are auxin-independent repressors (Tiwari *et al.*, 2003; Kato *et al.*, 2015, 2020), having unique motif preferences could dictate certain target genes free from A/B competition, and are therefore either generally activated or repressed.

Early work identified DR5 and IR7 motifs to be effective auxin-inducible reporters (Ulmasov *et al.*, 1997a, b). In 2016, the DNA affinity purification and sequencing (DAP-seq) method was developed for Arabidopsis transcription factors. In DAP-seq, genomic DNA is mixed with a transcription factor attached to magnetic beads that allows a ‘pull-down’ of the most strongly bound genomic regions, which yields both the genomic location of binding and the DNA-binding motifs for the transcription factor (O’Malley *et al.*, 2016). Recently, studies with this method have yielded the first comprehensive overview of genome-wide DNA binding by ARFs, revealing both overlapping and distinct motif preferences for A-ARFs and B-ARFs (O’Malley *et al.*, 2016; Galli *et al.*, 2018; Stigliani *et al.*, 2019). In both Arabidopsis and maize, A- and B-ARFs can bind IR7/8 motifs, while A-ARFs are additionally capable of binding to several DR and ER motifs. While there are clear differences between the DNA binding specificities of A- and B-classes of ARFs, relatively minor differences exist within each class. One caveat is that in DAP-seq, transcription factors are offered genomic DNA fragments devoid of histones, leading to binding events also occurring within regions normally inaccessible in a physiological context, highlighted by a low overlap of binding events between DAP-seq and ChIP-seq experiments (O’Malley *et al.*, 2016). In maize, only 5–25% of DAP-seq ARF binding events occur within open chromatin regions (Galli *et al* 2018). However, when combining DAP-seq with gene expression data in maize, it appeared that auxin-activated genes either have a sole A-ARF binding event, or shared A-ARF and B-ARF events, but never a B-ARF-only event (Galli *et al.*, 2018). This matches with data obtained in Arabidopsis, where promoters of auxin-activated genes are enriched for both binding events and motifs that are targeted primarily by A-ARFs (see below) (Stigliani *et al.*, 2019; Freire-Rios *et al.*, 2020). In other words, genes that are auxin activated will be bound by A-ARFs alone or by competing A-ARFs and B-ARFs, whereas auxin-repressed genes are solely bound by B-ARFs. Hence, the different conformations of tandem elements represent an additional layer of complexity to ARF-mediated transcriptional regulation.

### Inverted repeats

The IR7/8 conformation probably represents the canonical ARF competition model observed in Marchantia: A- and B-ARFs from Marchantia and Arabidopsis are able to bind this conformation, although B-ARFs bind with slightly lower affinity than A-ARFs (Fig. 3B); consistently, both auxin-activated and auxin-repressed genes in Arabidopsis are associated with these motifs (Boer *et al.*, 2014; Stigliani *et al.*, 2019; Freire-Rios *et al.*, 2020; Kato *et al.*, 2020; Fontana *et al.*, 2023). In agreement, DAP-seq binding events containing these motifs are enriched when these events are filtered by auxin-responsive genes, both in maize and in Arabidopsis (Galli *et al.*, 2018; Stigliani *et al.*, 2019). Single-molecule *in vitro* ARF–DNA binding assays suggest two different modes of binding: for B-class ARFs (AtARF1 and AtARF2), both the DBD and the PB1 domain facilitate cooperative homodimerization. Monomeric ARFs have a low affinity for DNA, and quickly dimerize via the DBD and the PB1 domain. Once dimerized, they bind IR7 tandem motifs, forming a more stable complex. However, this stabilization relies on the cooperative action of the DBD and PB1, as removing the PB1 leads to AtARF2 being primarily present as monomers in solution (Fontana *et al.*, 2022, 2023). This dual mode of cooperative binding probably holds true for other ARFs as well (Ulmasov *et al.*, 1999b). For the A-class AtARF5, even though the AtARF5 DBDs are present primarily as monomers in solution, the DBD itself is sufficient for strong dimerization and DNA binding, relying less on the cooperative action of the PB1 domain (Fontana *et al.*, 2022, 2023). The ARF5 PB1 domain has a higher affinity towards Aux/IAA than for homodimerization (Kim, 1997; Ouellet *et al.*, 2001; Hamann *et al.*, 2002; Muto *et al.*, 2006; Li *et al.*, 2011; Kim *et al.*, 2020; Fontana *et al.*, 2023), which matches with its primary function of being an auxin-dependent (through recruiting Aux/IAA) transcriptional activator (Tiwari *et al.*, 2003). Physiological evidence supports the importance of cooperative dimerization for proper ARF functioning: *AtTMO5* (itself a direct target of AtARF5) and *AtIAA19* promoters each have an IR motif that is only functional if an ARF dimer binds (Freire-Rios *et al.*, 2020). In line with this, *AtARF5* mutants in the dimerization domain of the DBD are unable to rescue an *Atarf5/mp* mutant (Boer *et al.*, 2014).

The crystal structures of AtARF1 (4ldx, 6ycq; Boer *et al.*, 2014; Freire-Rios *et al.*, 2020) and MpARF2 (6sdg; Kato *et al.*, 2020) DBDs revealed the binding mode on IR7 motifs. The DBD structure can be divided into three subdomains (Fig. 1B). The B3 domain, discussed above, is tethered by a single α-helix (α1) underneath a fold composed of the other two subdomains: the dimerization domain (DD) and the ancillary domain (AD). The DD face is composed of an α-helix (αD) that facilitates dimerization with the mirrored helix αD of the other monomer, while simultaneously a dimerization loop is inserted into a groove of the opposite monomer, leading to dimerization in a head-to-head orientation. The AD is located at the end of the DBD in the gene, and occupies the tail position in the structure of the dimer (Boer *et al.*, 2014). Upon dimer binding to the IR7 DNA, the DNA is slightly contorted and the B3 domains twist on helix α1 to enable placement of the two DNA-binding loops (discussed above). Even though AtARF1 and MpARF2 have had 500 million years of independent parallel evolution, their DNA-bound, dimerized structures are essentially the same (Boer *et al.*, 2014; Freire-Rios *et al.*, 2020; Kato *et al.*, 2020), suggesting a strong evolutionary pressure to maintain the ARF fold as it is.

Besides IR7/8, other IR motifs are likely to be selected by ARFs but are possibly rare. In carrot cells, promoters with IR7/8 are strongly auxin inducible, while IR5/6/9 are weakly inducible (Ulmasov *et al.*, 1997a). In surface plasmon resonance (SPR) analysis, a method for quantifying the affinity of two interacting molecules, including transcription factor DNA binding affinities, AtARF1 (B) was found to be restricted to IR7/8 motifs, while AtARF5 (A-class) could bind IR5, IR6, or IR9 motifs, although with lower affinity than IR7/8 (Boer *et al.*, 2014). Given how the B3 domains twist on α1 to adapt to IR7 DNA, it is plausible that, compared with B-ARFs, more intrinsic flexibility within A-ARFs in the helix α1 region could accommodate different IR conformations other than IR7/8. This model was termed the ‘caliper model’ by Boer *et al* (2014) (Fig. 3C) and could offer an explanation for why some ARFs can, and others cannot, functionally complement each other (Hardtke *et al.*, 2004; Nagpal *et al.*, 2005; Okushima *et al.*, 2005; Weijers *et al.*, 2005; Li *et al.*, 2006; Rademacher *et al.*, 2011, 2012; Kato *et al.*, 2020). However, whether the caliper model extends beyond AtARF1 and AtARF5 is unknown. So far, *in vivo* only IR9 motifs have been identified, in promoters of *AtLFY*, *AtTMO3*, and *AtIAA19* (Rademacher *et al.*, 2011; Yamaguchi *et al.*, 2013; Freire-Rios *et al.*, 2020). It is therefore likely that when it comes to IR motifs, motifs other than IR7/8 are likely to be functional, but since they have not been identified with either DAP-seq or promoter meta-analyses (Galli *et al.*, 2018; Freire-Rios *et al.*, 2020), these other motifs are likely to be rare.

### Direct repeats

DR motifs appear to be conformations that are relatively specific to A-ARFs and auxin-dependent activation (Galli *et al.*, 2018; Stigliani *et al.*, 2019; Freire-Rios *et al.*, 2020). DR5 became the *de facto* auxin response reporter based on its strong auxin inducibility (Ulmasov *et al.*, 1997b), well before ARF motif conformation spacing was studied and DR5 motifs were identified in auxin-regulated genes (Lieberman-Lazarovich *et al.*, 2019). A possible explanation for why DR5 is an effective reporter can be found in the most recent DAP-seq data, where only A-ARFs appear to bind DR4/5 motifs (O’Malley *et al.*, 2016; Galli *et al.*, 2018; Stigliani *et al.*, 2019). In maize, auxin-activated genes are associated with A-ARF binding events, and in Arabidopsis, auxin-activated genes are associated with AtARF5 binding to DR5 motifs (Galli *et al.*, 2018; Freire-Rios *et al.*, 2020). These results suggest that only A-ARFs and not B-ARFs are capable of binding to DR5 *in vivo*. *In vitro*, however, both classes are able to bind DR5, albeit with much weaker affinities than to IR7 or IR8 motifs (Fig. 3B) (Kato *et al.*, 2020; Freire-Rios *et al.*, 2020). If we speculate, it might be possible that B-ARF affinities are so low that DR5 occupancy by B-ARFs is negligible or that B-ARFs are titrated from DR5 sites by high-affinity (IR7/8) sites, such that A-ARFs can more easily bind DR5 motifs and effectively turn them into a net strong auxin-activated motif (Freire-Rios *et al.*, 2020). Structurally, we can only speculate as to how DNA binding is achieved. A DR5 motif composed of two TGTC repeats becomes auxin unresponsive when either element is mutated (Liu *et al.*, 1994; Ulmasov *et al.*, 1997b), suggesting that a dimer binds cooperatively. Furthermore, binding analyses were performed with ARF DBDs only, indicating that the DBD is sufficient for binding DR5 motifs (Freire-Rios *et al.*, 2020). The 5 bp spacing in DR5 is a full DNA helix twist, suggesting that the dimer binds on the same plane. This would place the DBDs in a head-to-tail orientation; that is, with the dimerization α-helix (αD) and AD (Fig. 1B) potentially interfacing each other. DR4 and DR6 would place DBDs slightly off kilter; they are only weakly auxin inducible in carrots (Ulmasov *et al.*, 1997b), and only DR4 is bound by maize and Arabidopsis A-ARFs *in vivo* (Galli *et al.*, 2018; Stigliani *et al.*, 2019). All in all, while the DR5 reporter celebrates its 25th anniversary, this motif conformation still harbours secrets.

### Everted repeats

ER motifs remain an enigma, as no functional sites have been identified *in vivo* nor has their structure been solved, yet ER motifs are enriched in DAP-seq experiments. ER motifs are only weakly enriched in promoters of auxin-regulated genes (Freire-Rios *et al.*, 2020). Still, both A- and B-ARFs can bind ER motifs *in vitro*, although their spacing preference is family specific (Galli *et al.*, 2018; Stigliani *et al.*, 2019). For AtARF5, an ER0 motif was enriched, which would have one of the monomers on the complete 180° opposite side of the DNA helix. This orientation would preclude dimerization via the DBD for the ARFs, therefore relying on the PB1 domain, to facilitate DNA binding. AtARF5 also binds ER13, placing the ARFs on the same plane of the DNA helix, in a tail-to-tail (AD to AD) conformation (Stigliani *et al.*, 2019). Given that ER motifs are enriched in DAP-seq, it at least indicates the presence of such conformations in the genome and that they can be bound by ARFs *in vitro*, but their functional importance is yet to be proven *in vivo*.

## Control of ARF localization and stability

ARFs are the pivot point in auxin-dependent gene regulation. As discussed above, intrinsic properties provide DNA binding specificity, while Aux/IAA association determines activity and A/B competition defines auxin sensitivity. Within this relatively simple response system, behaviour is dictated by biochemical interaction parameters. Biological interactions though are a function of both affinity and concentration, and, therefore, any mechanisms that control (local) protein concentration can impact ARF action (Kribelbauer *et al.*, 2019).

ARF PB1 crystal structures revealed that these domains can oligomerize (Han *et al.*, 2014; Korasick *et al.*, 2014; Nanao *et al.*, 2014; Kim *et al.*, 2020). The PB1 domain is composed of a positively charged face (mainly due to an invariant lysine) and a negatively charged face (named OPCA after OPR, PC, and AID) (Ponting *et al.*, 2002). Two PB1 domains will interact in a head-to-tail manner, and more PB1 domains could oligomerize on either side. Oligomerization is clearly a mechanism to create higher local protein concentration, perhaps to enhance binding to low-affinity sites, or to cause transcriptional bursts (Kribelbauer *et al.*, 2019; Wei *et al.*, 2020). However, it is not yet clear if ARF oligomerization enhances DNA binding, as additional oligomers do not enhance the binding on a single IR7 motif (i.e. two TGTCGG elements) *in vitro* (Fontana *et al.*, 2023), although oligomerization does enhance activation in a heterologous yeast system (Pierre-Jerome *et al.*, 2016). In DAP-seq experiments, both A-ARFs and B-ARFs bind to motifs that have a spacing that is too large for a single dimer to bind, and it was proposed that they are bound by oligomers (Galli *et al.*, 2018; Stigliani *et al.*, 2019). Outside of the nucleus, oligomerization appears to function in sequestering A-ARFs into the cytoplasm: AtARF19 accumulates in oligomerization-driven cytoplasmic condensates, in particular in differentiated root cells. The condensation appears to involve the intrinsically disordered—prion-like—MR, but critically requires interactions through the PB1 domain. Mutating the charged faces of the AtARF19 PB1 domain restores nuclear localization, prevents condensation, and leads to hyperactivity of the ARF (Powers *et al.*, 2019). Whether oligomerization is involved in DNA binding, controlling nuclear levels of ARFs, or if it serves a different function will be questions for the future.

Genome-wide, TGTCNN elements are widespread, yet only a fraction of them are bound by ARFs. ARF dimerization is one way to add specificity for DNA binding, but like any equilibrium, monomeric species exist in the cell nucleus with an inherent capability to bind a single TGTCNN motif, that would be expected to occur once every 256 bp by chance. Recently, it has been suggested that some of these monomeric forms are actively degraded to prevent monomeric binding that could trigger undesired transcriptional changes. In Marchantia, both MpARF1 and MpARF2 are degraded via the proteasomal machinery. The MpARF2 degron motif overlaps with the dimerization helix (αD, Fig. 1B) of the DBD, and is probably unavailable upon dimerization, thus preventing degradation of dimeric MpARF2. Non-dimerizing mutants are rapidly degraded; however, when these monomeric species are additionally mutated in the degron motif, the resulting plants show serious defects in thallus development (Das *et al.*, 2022, Preprint). This suggests that monomeric species can cause significant transcriptional misregulation in the cell, and degradation of these monomeric species could be a way to ensure the correct gene targets are regulated.

There is a precedent for ARF protein degradation. AtARF1 was shown to be an unstable protein with a half-life of ~3 h (Salmon *et al.*, 2008; Lakehal *et al.*, 2019). This instability was shown to depend on proteasomal degradation, but not on the Skp, Cullin, F-box-containing complex (SCF) subunit CULLIN 1. The biological relevance of degradation has not yet been established, but there is clearly scope for active control of AtARF1 accumulation. Likewise, AtARF2 appears to be subject to proteasomal degradation: the hormone gibberellin (GA) promotes ARF2 accumulation, probably by preventing its degradation (Richter *et al.*, 2013). AtARF1, AtARF6, AtARF8, and AtARF17 are all actively degraded via the proteasome in Arabidopsis protoplasts (Lakehal *et al.*, 2019). More recently, it was reported that the E3 ligase AFF1 mediates degradation of AtARF7 and AtARF19 in the Arabidopsis root, acting as a mechanism for fine-tuning ARF levels within a cell (Jing *et al.*, 2022). Mutants in the E3 ligase have a weak but significant phenotype in auxin response, which suggests a biological role for ARF degradation. Thus, with these examples from Arabidopsis A-class (AtARF7/19) and B-class (AtARF1/2) and both A- and B-class Marchantia ARFs, it is emerging that ARF degradation may be a common principle. This raises several intriguing questions. Are all ARFs degraded via E3 ligase-mediated degradation? Are there multiple mechanisms? Where are the degron motifs located? The biological importance of ARF degradation has been suggested in both Marchantia and Arabidopsis and will be likely to lead to many exciting findings.

## Open and pressing questions

Recent work in Marchantia has revealed what is probably the minimal, ancestral-like state of auxin regulation by the ARFs, wherein the A-ARF is auxin sensitive and the A- and B-ARFs are competing for the same motifs. It is then the stoichiometry of the two types of ARFs that dictates the auxin sensitivity of a cell. However, no matter the simplicity of the Marchantia system, there are still many questions that remain to be answered. Given that, compared with B-ARFs (only IR motifs), angiosperm A-ARFs can probably bind a wider variety of tandem repeat motifs (DRs, ERs, and IRs), does this also occur in MpARF1 and MpARF2? Does MpARF1 have a caliper capacity like AtARF5 to extended IR motifs? How much overlap exists between MpARF1 and MpARF2 DNA-binding sites *in vivo*? Answering these questions will provide the basic framework for how A- and B-ARFs work at the simplest level and will allow us to investigate the unique specificities that ARFs acquired during sub- and neofunctionalization of the paralogues existing in angiosperms.

ARFs most probably originated in a common ancestor to all streptophytes, but we do not know their ancestral function or characteristics. Only one algal ARF has been studied for its biochemical properties and, while it can bind TGTCNN motifs, its spacing preferences and phylogeny suggest that it is related to C-ARFs rather than to the A/B-ARFs (Martin-Arevalillo *et al.*, 2019; Carrillo-Carrasco *et al.*, 2023). Since evidence suggests that C-ARFs are not involved in the transcriptional response to auxin, at least in Marchantia (Flores-Sandoval *et al.*, 2018b; Mutte *et al.*, 2018; Kato *et al.*, 2020), it remains an open question regarding the function of binding to, and potentially competing on, TGTCNN motifs by C-ARFs. It will be interesting to find out if the DBDs of algal ARFs share some or all their properties with land plant ARFs. Do these ARFs dimerize? Did the ancestral ARF have features of A-, B-, and/or C-ARFs or are their characteristics entirely unrelated to land plant ARFs? ARFs were already separated into three clades in the last land plant common ancestor, with Marchantia having a minimal NAP system that could be reminiscent of how an ancestral system would have functioned. Having such a simple, yet essential system would place evolutionary constraints on mutations that would disrupt the carefully balanced stoichiometry of MpARF1 and MpARF2. That begs the question: can we find other bryophytes with a similar minimal system, and do they share the same features as the ARFs in Marchantia?

ARF families expanded following whole-genome duplications in seed plants, freeing them from the evolutionary constraints of the minimalistic system and allowing for neo- and subfunctionalization. In Arabidopsis and maize, ARFs have unique expression domains, not all ARFs are able to replace each other, and the greater phylogenetic distance between ARFs, the less overlap in genomic binding profiles they have. The opposite can also be true: more closely related ARFs act redundantly (AtARF7 and AtARF19) and share more similar DNA binding patterns (ZmARF4 and ZmARF29). These additional complexities make studying ARFs in flowering plants challenging. Despite relatively good understanding of the generic mechanisms underlying auxin response, there is a pressing scarcity of insight into the actions of individual ARFs in their functional context. Improvements in single-cell resolution methods for DNA-binding (CUT&RUN) (Skene and Henikoff, 2017), (single-cell) transcriptomics (Kolodziejczyk *et al.*, 2015; Lavrekha *et al.*, 2022), and protein–protein interactions (proximity labelling techniques) (Cortal *et al.*, 2021) may offer the resolution needed to study ARF function at the granularity necessary to dissect their unique functions in growth and development. In parallel, further improvements in statistical modelling (Lai *et al.*, 2019), and machine learning tools (Alipanahi *et al.*, 2015), should enable us to identify not only the major patterns in the data (IR7/IR8, TGTCGG), but also the rare events (IR9, TGTCTC) that are likely to be highly relevant in specific contexts, as seen from unique expression domains of different TGTCNN reporters in Arabidopsis roots. These methods would enable us to answer questions such as: what are the ARF caliper potentials? Even though all ARFs favour TGTCGG elements, do their preferences differ for medium affinity elements? What motifs are enriched in auxin-responsive genes on a single-cell level?

ARF DBD and PB1 crystal structures have enabled linking functions to structures for dimerization, DNA binding, and degradation. However, many questions remain: how do A-ARFs bind DR5? Can crystal structures of A-ARFs with IR5 or IR9 explain the caliper model? Regarding the TGTCNN specificity, while the DNA-binding residues are conserved, there is variation in the B3 domain itself between ARF paralogues and orthologues. Can this variation affect DNA binding or would all B3 domains target essentially the same motifs?

Over the past three decades, the scientific community has unravelled a simple nuclear auxin signalling pathway that boils down to ARFs deciding which genes need to be auxin regulated. This simplicity is deceptive, as each ARF seems to have has its own peculiarities. Nevertheless, many secrets of the ARFs remain to be revealed, a challenge our research community is ready to tackle.

## References

[CIT0001] Alipanahi B , Delong A, Weirauch MT, Frey BJ. 2015. Predicting the sequence specificities of DNA- and RNA-binding proteins by deep learning. Nature Biotechnology 33, 831–838. 10.1038/nbt.3300 26213851

[CIT0002] Amoutzias GD , Robertson DL, Van de Peer Y, Oliver SG. 2008. Choose your partners: dimerization in eukaryotic transcription factors. Trends in Biochemical Sciences 33, 220229. 10.1016/j.tibs.2008.02.002 18406148

[CIT0003] Boer DR , Freire-Rios A, van den Berg WA, et al. 2014. Structural basis for DNA binding specificity by the auxin-dependent ARF transcription factors. Cell 156, 577–589. 24485461 10.1016/j.cell.2013.12.027

[CIT0004] Bowman JL , Kohchi T, Yamato KT, et al. 2017. Insights into land plant evolution garnered from the *Marchantia polymorpha* genome. Cell 171, 287–304 e215. 28985561 10.1016/j.cell.2017.09.030

[CIT0005] Carrillo-Carrasco VP , Hernandez-Garcia J, Mutte SK, Weijers D. 2023. The birth of a giant: evolutionary insights into the origin of auxin responses in plants. The EMBO Journal 42, e113018. 36786017 10.15252/embj.2022113018 PMC10015382

[CIT0006] Cherenkov P , Novikova D, Omelyanchuk N, Levitsky V, Grosse I, Weijers D, Mironova V. 2018. Diversity of cis-regulatory elements associated with auxin response in *Arabidopsis thaliana*. Journal of Experimental Botany 69, 329–339. 28992117 10.1093/jxb/erx254 PMC5853796

[CIT0007] Cortal A , Martignetti L, Six E, Rausell A. 2021. Gene signature extraction and cell identity recognition at the single-cell level with Cell-ID. Nature Biotechnology 39, 1095–1102. 10.1038/s41587-021-00896-6 33927417

[CIT0008] Das S , de Roij M, Bellows S, Kohlen W, Farcot E, Weijers D, Borst JW. 2022. Selective degradation of ARF monomers controls auxin response in Marchantia. bioRxiv doi: 10.1101/2022.11.04.515187. [Preprint].

[CIT0009] Donner TJ , Sherr I, Scarpella E. 2009. Regulation of preprocambial cell state acquisition by auxin signaling in Arabidopsis leaves. Development 136, 3235–3246. 19710171 10.1242/dev.037028

[CIT0010] Flores-Sandoval E , Eklund DM, Bowman JL. 2015. A simple auxin transcriptional response system regulates multiple morphogenetic processes in the liverwort *Marchantia polymorpha*. PLoS Genetics 11, e1005207. 26020649 10.1371/journal.pgen.1005207 PMC4447368

[CIT0011] Flores-Sandoval E , Eklund DM, Hong SF, et al. 2018a. Class C ARFs evolved before the origin of land plants and antagonize differentiation and developmental transitions in *Marchantia polymorpha*. New Phytologist 218, 1612–1630. 29574879 10.1111/nph.15090

[CIT0012] Flores-Sandoval E , Romani F, Bowman JL. 2018b. Co-expression and transcriptome analysis of *Marchantia polymorpha* transcription factors supports class C ARFs as independent actors of an ancient auxin regulatory module. Frontiers in Plant Science 9, 1345. 30327658 10.3389/fpls.2018.01345 PMC6174852

[CIT0013] Fontana M , Ivanovaite S, Lindhoud S, van der Wijk E, Mathwig K, Berg WVD, Weijers D, Hohlbein J. 2022. Probing DNA–transcription factor interactions using single-molecule fluorescence detection in nanofluidic devices. Advanced Biology 6, e2100953. 34472724 10.1002/adbi.202100953

[CIT0014] Fontana M , Roosjen M, Crespo Garcia I, van den Berg W, Malfois M, Boer R, Weijers D, Hohlbein J. 2023. Cooperative action of separate interaction domains promotes high-affinity DNA binding of *Arabidopsis thaliana* ARF transcription factors. Proceedings of the National Academy of Sciences, USA 120, e2219916120. 10.1073/pnas.2219916120 PMC10089223 36881630

[CIT0015] Franco-Zorrilla JM , Lopez-Vidriero I, Carrasco JL, Godoy M, Vera P, Solano R. 2014. DNA-binding specificities of plant transcription factors and their potential to define target genes. Proceedings of the National Academy of Sciences, USA 111, 2367–2372. 10.1073/pnas.1316278111 PMC3926073 24477691

[CIT0016] Freire-Rios A , Tanaka K, Crespo I, et al. 2020. Architecture of DNA elements mediating ARF transcription factor binding and auxin-responsive gene expression in Arabidopsis. Proceedings of the National Academy of Sciences, USA 117, 24557–24566. 10.1073/pnas.2009554117 PMC7533888 32929017

[CIT0017] Galli M , Khakhar A, Lu Z, Chen Z, Sen S, Joshi T, Nemhauser JL, Schmitz RJ, Gallavotti A. 2018. The DNA binding landscape of the maize AUXIN RESPONSE FACTOR family. Nature Communications 9, 4526. 10.1038/s41467-018-06977-6 PMC6207667 30375394

[CIT0018] Hamann T , Benkova E, Baurle I, Kientz M, Jurgens G. 2002. The Arabidopsis BODENLOS gene encodes an auxin response protein inhibiting MONOPTEROS-mediated embryo patterning. Genes & Development 16, 1610–1615. 12101120 10.1101/gad.229402 PMC186366

[CIT0019] Han M , Park Y, Kim I, Kim EH, Yu TK, Rhee S, Suh JY. 2014. Structural basis for the auxin-induced transcriptional regulation by Aux/IAA17. Proceedings of the National Academy of Sciences, USA 111, 18613–18618. 10.1073/pnas.1419525112 PMC4284525 25512488

[CIT0020] Hardtke CS , Ckurshumova W, Vidaurre DP, Singh SA, Stamatiou G, Tiwari SB, Hagen G, Guilfoyle TJ, Berleth T. 2004. Overlapping and non-redundant functions of the Arabidopsis auxin response factors MONOPTEROS and NONPHOTOTROPIC HYPOCOTYL 4. Development 131, 1089–1100. 14973283 10.1242/dev.00925

[CIT0021] Huang KL , Ma GJ, Zhang ML, et al. 2018. The ARF7 and ARF19 transcription factors positively regulate PHOSPHATE STARVATION RESPONSE1 in Arabidopsis roots. Plant Physiology 178, 413–427. 30026290 10.1104/pp.17.01713 PMC6130041

[CIT0022] Jing H , Korasick DA, Emenecker RJ, Morffy N, Wilkinson EG, Powers SK, Strader LC. 2022. Regulation of AUXIN RESPONSE FACTOR condensation and nucleo-cytoplasmic partitioning. Nature Communications 13, 4015. 10.1038/s41467-022-31628-2 PMC9273615 35817767

[CIT0023] Kato H , Ishizaki K, Kouno M, Shirakawa M, Bowman JL, Nishihama R, Kohchi T. 2015. Auxin-mediated transcriptional system with a minimal set of components is critical for morphogenesis through the life cycle in *Marchantia polymorpha*. PLoS Genetics 11, e1005084. 26020919 10.1371/journal.pgen.1005084 PMC4447296

[CIT0024] Kato H , Kouno M, Takeda M, Suzuki H, Ishizaki K, Nishihama R, Kohchi T. 2017. The roles of the sole activator-type auxin response factor in pattern formation of *Marchantia polymorpha*. Plant and Cell Physiology 58, 1642–1651. 29016901 10.1093/pcp/pcx095

[CIT0025] Kato H , Nishihama R, Weijers D, Kohchi T. 2018. Evolution of nuclear auxin signaling: lessons from genetic studies with basal land plants. Journal of Experimental Botany 69, 291–301. 28992186 10.1093/jxb/erx267

[CIT0026] Kato H , Mutte SK, Suzuki H, et al. 2020. Design principles of a minimal auxin response system. Nature Plants 6, 473–482. 32415296 10.1038/s41477-020-0662-y

[CIT0027] Kim J. 1997. Protein–protein interactions among the Aux/IAA proteins. Proceedings of the National Academy of Sciences, USA 94, 1178611791. 10.1073/pnas.94.22.11786 PMC23574 9342315

[CIT0028] Kim Y , Park C, Cha S, Han M, Ryu KS, Suh JY. 2020. Determinants of PB1 domain interactions in auxin response factor ARF5 and repressor IAA17. Journal of Molecular Biology 432, 4010–4022. 32305460 10.1016/j.jmb.2020.04.007

[CIT0029] Kolodziejczyk AA , Kim JK, Svensson V, Marioni JC, Teichmann SA. 2015. The technology and biology of single-cell RNA sequencing. Molecular Cell 58, 610–620. 26000846 10.1016/j.molcel.2015.04.005

[CIT0030] Korasick DA , Westfall CS, Lee SG, Nanao MH, Dumas R, Hagen G, Guilfoyle TJ, Jez JM, Strader LC. 2014. Molecular basis for AUXIN RESPONSE FACTOR protein interaction and the control of auxin response repression. Proceedings of the National Academy of Sciences, USA 111, 54275432. 10.1073/pnas.1400074111 PMC3986151 24706860

[CIT0031] Kribelbauer JF , Rastogi C, Bussemaker HJ, Mann RS. 2019. Low-affinity binding sites and the transcription factor specificity paradox in eukaryotes. Annual Review of Cell and Developmental Biology 35, 357–379. 10.1146/annurev-cellbio-100617-062719 PMC6787930 31283382

[CIT0032] Lai X , Stigliani A, Vachon G, Carles C, Smaczniak C, Zubieta C, Kaufmann K, Parcy F. 2019. Building transcription factor binding site models to understand gene regulation in plants. Molecular Plant 3, 743–763. 10.1016/j.molp.2018.10.010 30447332

[CIT0033] Lakehal A , Chaabouni S, Cavel E, et al. 2019. A molecular framework for the control of adventitious rooting by TIR1/AFB2–Aux/IAA-dependent auxin signaling in Arabidopsis. Molecular Plant 12, 1499–1514. 31520787 10.1016/j.molp.2019.09.001

[CIT0034] Lavrekha VV , Levitsky VG, Tsukanov AV, Bogomolov AG, Grigorovich DA, Omelyanchuk N, Ubogoeva EV, Zemlyanskaya EV, Mironova V. 2022. CisCross: a gene list enrichment analysis to predict upstream regulators in *Arabidopsis thaliana*. Frontiers in Plant Science 13, 942710. 36061801 10.3389/fpls.2022.942710 PMC9434332

[CIT0035] Lavy M , Prigge MJ, Tao S, Shain S, Kuo A, Kirchsteiger K, Estelle M. 2016. Constitutive auxin response in *Physcomitrella* reveals complex interactions between Aux/IAA and ARF proteins. eLife 5, e13325. 27247276 10.7554/eLife.13325 PMC4889330

[CIT0036] Li J , Dai X, Zhao Y. 2006. A role for auxin response factor 19 in auxin and ethylene signaling in Arabidopsis. Plant Physiology 140, 899–908. 16461383 10.1104/pp.105.070987 PMC1400570

[CIT0037] Li JF , Bush J, Xiong Y, Li L, McCormack M. 2011. Large-scale protein–protein interaction analysis in Arabidopsis mesophyll protoplasts by split firefly luciferase complementation. PLoS One 6, e27364. 22096563 10.1371/journal.pone.0027364 PMC3212559

[CIT0038] Liao CY , Smet W, Brunoud G, Yoshida S, Vernoux T, Weijers D. 2015. Reporters for sensitive and quantitative measurement of auxin response. Nature Methods 12, 207–210. 25643149 10.1038/nmeth.3279 PMC4344836

[CIT0039] Lieberman-Lazarovich M , Yahav C, Israeli A, Efroni I. 2019. Deep conservation of cis-element variants regulating plant hormonal responses. The Plant Cell 31, 2559–2572. 31467248 10.1105/tpc.19.00129 PMC6881130

[CIT0040] Liu ZB , Ulmasov T, Shi X, Hagen G, Guilfoyle TJ. 1994. Soybean GH3 promoter contains multiple auxin-inducible elements. The Plant Cell 6, 645–657. 8038604 10.1105/tpc.6.5.645 PMC160465

[CIT0041] Martin-Arevalillo R , Thevenon E, Jegu F, Vinos-Poyo T, Vernoux T, Parcy F, Dumas R. 2019. Evolution of the auxin response factors from charophyte ancestors. PLoS Genetics 15, e1008400. 31553720 10.1371/journal.pgen.1008400 PMC6797205

[CIT0042] Muto H , Nagao I, Demura T, Fukuda H, Kinjo M, Yamamoto KT. 2006. Fluorescence cross-correlation analyses of the molecular interaction between an Aux/IAA protein, MSG2/IAA19, and protein–protein interaction domains of auxin response factors of Arabidopsis expressed in HeLa cells. Plant and Cell Physiology 47, 1095–1101. 16854942 10.1093/pcp/pcj080

[CIT0043] Mutte SK , Kato H, Rothfels C, Melkonian M, Wong GK, Weijers D. 2018. Origin and evolution of the nuclear auxin response system. eLife 7, e33399. 29580381 10.7554/eLife.33399 PMC5873896

[CIT0044] Nagpal P , Ellis CM, Weber H, et al. 2005. Auxin response factors ARF6 and ARF8 promote jasmonic acid production and flower maturation. Development 132, 4107–4118. 16107481 10.1242/dev.01955

[CIT0045] Nanao MH , Vinos-Poyo T, Brunoud G, et al. 2014. Structural basis for oligomerization of auxin transcriptional regulators. Nature Communications 5, 3617. 10.1038/ncomms4617 24710426

[CIT0046] Narise T , Kobayashi K, Baba S, Shimojima M, Masuda S, Fukaki H, Ohta H. 2010. Involvement of auxin signaling mediated by IAA14 and ARF7/19 in membrane lipid remodeling during phosphate starvation. Plant Molecular Biology 72, 533–544. 20043234 10.1007/s11103-009-9589-4

[CIT0047] Okushima Y , Overvoorde PJ, Arima K, et al. 2005. Functional genomic analysis of the AUXIN RESPONSE FACTOR gene family members in *Arabidopsis thaliana*: unique and overlapping functions of ARF7 and ARF19. The Plant Cell 17, 444–463. 15659631 10.1105/tpc.104.028316 PMC548818

[CIT0048] O’Malley RC , Huang SC, Song L, Lewsey MG, Bartlett A, Nery JR, Galli M, Gallavotti A, Ecker JR. 2016. Cistrome and epicistrome features shape the regulatory DNA landscape. Cell 165, 1280–1292. 27203113 10.1016/j.cell.2016.04.038 PMC4907330

[CIT0049] Ouellet F , Overvoorde PJ, Theologis A. 2001. IAA17/AXR3: biochemical insight into an auxin mutant phenotype. The Plant Cell 13, 829–841. 11283339 10.1105/tpc.13.4.829 PMC135541

[CIT0050] Pierre-Jerome E , Moss BL, Lanctot A, Hageman A, Nemhauser JL. 2016. Functional analysis of molecular interactions in synthetic auxin response circuits. Proceedings of the National Academy of Sciences, USA 113, 1135411359. 10.1073/pnas.1604379113 PMC5056036 27647902

[CIT0051] Ponting CP , Ito T, Moscat J, Diaz-Meco MT, Inagaki F, Sumimoto H. 2002. OPR, PC and AID: all in the PB1 family. Trends in Biochemical Science 27, 10. 10.1016/s0968-0004(01)02006-0 11796218

[CIT0052] Powers SK , Holehouse AS, Korasick DA, et al. 2019. Nucleo-cytoplasmic partitioning of ARF proteins controls auxin responses in *Arabidopsis thaliana*. Molecular Cell 76, 177–190 e175. 31421981 10.1016/j.molcel.2019.06.044 PMC6778021

[CIT0053] Rademacher EH , Lokerse AS, Schlereth A, et al. 2012. Different auxin response machineries control distinct cell fates in the early plant embryo. Developmental Cell 22, 211–222. 22264733 10.1016/j.devcel.2011.10.026

[CIT0054] Rademacher EH , Moller B, Lokerse AS, Llavata-Peris CI, van den Berg W, Weijers D. 2011. A cellular expression map of the Arabidopsis AUXIN RESPONSE FACTOR gene family. The Plant Journal 68, 597–606. 21831209 10.1111/j.1365-313X.2011.04710.x

[CIT0055] Richter R , Behringer C, Zourelidou M, Schwechheimer C. 2013. Convergence of auxin and gibberellin signaling on the regulation of the GATA transcription factors GNC and GNL in *Arabidopsis thaliana*. Proceedings of the National Academy of Sciences, USA 110, 13192–13197. 10.1073/pnas.1304250110 PMC3740866 23878229

[CIT0056] Salmon J , Ramos J, Callis J. 2008. Degradation of the auxin response factor ARF1. The Plant Journal 54, 118–128. 18088308 10.1111/j.1365-313X.2007.03396.x

[CIT0057] Skene PJ , Henikoff S. 2017. An efficient targeted nuclease strategy for high-resolution mapping of DNA binding sites. eLife 6, e21856. 28079019 10.7554/eLife.21856 PMC5310842

[CIT0058] Sloan J , Hakenjos JP, Gebert M, Ermakova O, Gumiero A, Stier G, Wild K, Sinning I, Lohmann JU. 2020. Structural basis for the complex DNA binding behavior of the plant stem cell regulator WUSCHEL. Nature Communications 11, 2223. 10.1038/s41467-020-16024-y PMC7203112 32376862

[CIT0059] Stigliani A , Martin-Arevalillo R, Lucas J, Bessy A, Vinos-Poyo T, Mironova V, Vernoux T, Dumas R, Parcy F. 2019. Capturing auxin response factors syntax using DNA binding models. Molecular Plant 12, 822–832. 30336329 10.1016/j.molp.2018.09.010

[CIT0060] Tan X , Calderon-Villalobos LIA, Sharon M, Zheng C, Robinson CV, Estelle M, Zheng N. 2007. Mechanism of auxin perception by the TIR1 ubiquitin ligase. Nature 446, 640–645. 17410169 10.1038/nature05731

[CIT0061] Tiwari SB , Hagen G, Guilfoyle T. 2003. The roles of auxin response factor domains in auxin-responsive transcription. The Plant Cell 15, 533–543. 12566590 10.1105/tpc.008417 PMC141219

[CIT0062] Tiwari SB , Wang XJ, Hagen G, Guilfoyle TJ. 2001. AUX/IAA proteins are active repressors, and their stability and activity are modulated by auxin. The Plant Cell 13, 2809–2822. 11752389 10.1105/tpc.010289 PMC139490

[CIT0063] Ulmasov T , Hagen G, Guilfoyle TJ. 1997a. ARF1, a transcription factor that binds to auxin response elements. Science 276, 1865–1868. 9188533 10.1126/science.276.5320.1865

[CIT0064] Ulmasov T , Hagen G, Guilfoyle TJ. 1999a. Activation and repression of transcription by auxin-response factors. Proceedings of the National Academy of Sciences, USA 96, 58445849. 10.1073/pnas.96.10.5844 PMC21948 10318972

[CIT0065] Ulmasov T , Hagen G, Guilfoyle TJ. 1999b. Dimerization and DNA binding of auxin response factors. The Plant Journal 19, 309–319. 10476078 10.1046/j.1365-313x.1999.00538.x

[CIT0066] Ulmasov T , Liu ZB, Hagen G, Guilfoyle TJ. 1995. Composite structure of auxin response elements. The Plant Cell 7, 1611–1623. 7580254 10.1105/tpc.7.10.1611 PMC161020

[CIT0067] Ulmasov T , Murfett J, Hagen G, Guilfoyle TJ. 1997b. Aux/IAA proteins repress expression of reporter genes containing natural and highly active synthetic auxin response elements. The Plant Cell 9, 1963–1971. 9401121 10.1105/tpc.9.11.1963 PMC157050

[CIT0068] Walcher CL , Nemhauser JL. 2012. Bipartite promoter element required for auxin response. Plant Physiology 158, 273–282. 22100645 10.1104/pp.111.187559 PMC3252081

[CIT0069] Wei MT , Chang YC, Shimobayashi SF, Shin Y, Strom AR, Brangwynne CP. 2020. Nucleated transcriptional condensates amplify gene expression. Nature Cell Biology 22, 1187–1196. 32929202 10.1038/s41556-020-00578-6

[CIT0070] Weijers D , Benkova E, Jager KE, Schlereth A, Hamann T, Kientz M, Wilmoth JC, Reed JW, Jurgens G. 2005. Developmental specificity of auxin response by pairs of ARF and Aux/IAA transcriptional regulators. The EMBO Journal 24, 1874–1885. 15889151 10.1038/sj.emboj.7600659 PMC1142592

[CIT0071] Weijers D , Wagner D. 2016. Transcriptional responses to the auxin hormone. Annual Review of Plant Biology 67, 539–574. 10.1146/annurev-arplant-043015-112122 26905654

[CIT0072] Xu N , Hagen G, Guilfoyle T. 1997. Multiple auxin response modules in the soybean SAUR 15A promoter. Plant Science 126, 193–201.

[CIT0073] Yamaguchi N , Wu M-F, Winter CM, Markus MC, Nole-Wilson S, Yamaguchi A, Coupland G, Krizek BA, Wagner D. 2013. A molecular framework for auxin-mediated initiation of flower primordia. Developmental Cell 24, 271–282. 23375585 10.1016/j.devcel.2012.12.017

[CIT0074] Zemlyanskaya EV , Wiebe DS, Omelyanchuk NA, Levitsky VG, Mironova VV. 2016. Meta-analysis of transcriptome data identified TGTCNN motif variants associated with the response to plant hormone auxin in *Arabidopsis thaliana* L. Journal of Bioinformatics and Computational Biology 14, 1641009. 27122321 10.1142/S0219720016410092

